# Reflections on: Lactate dynamics in cardiac surgery

**DOI:** 10.34172/jcvtr.025.33264

**Published:** 2025-12-17

**Authors:** Rohan Magoon

**Affiliations:** Department of Anaesthesia and Cardiac Anaesthesia, Atal Bihari Vajpayee Institute of Medical Sciences (ABVIMS) and Dr. Ram Manohar Lohia Hospital, Baba Kharak Singh Marg, New Delhi, INDIA

## Dear Editor,

 Hadipourzadeh, et al are congratulated for their retrospective research endeavor, studying the relationship between lactate levels and postoperative renal dysfunction, in 395 patients undergoing coronary artery bypass grafting surgery (CABG).^1^ However, the lactate dynamics can be intricate enough to warrant further discussion.

 Hyperlactatemia can effectively result from the anaerobic or aerobic pathways, as it relates to either an excessive production or a reduced clearance of lactate. Of note, the former manifests in nearly half of the cardiac surgical patients, wherein being multifactorial in etiology, hyperlactatemia might not necessarily be synonymous with an underlying hypoxic milieu.^2,3^ Emphasizing upon the same, a 2024 scoping review by de Castro Teixeira et al outline cardiopulmonary bypass (CPB) time, tissue hypoperfusion and, the use of vasoactive drugs and corticosteroids, as the major factors associated with lactate elevation in cardiac surgery, alongside suggesting relevant connections between perioperative hyperlactatemia (especially early-onset) and outcomes.^2^ Having said that, the steroid practice in the Hadipourzadeh et al study, remains to be clarified for the readers.^1^ Moreover, the authors happen to report the use of intraoperative hemodynamic infusions qualitatively without accounting for the corresponding doses.^1^ Literature exists to relate quantitative parameters such as vasoactive-inotropic score (VIS) with acute kidney injury (AKI) in surgical settings like the index study, as highlighted in a systematic review and meta-analysis by Sun, et al including a total of 58 studies and 29,920 patients, with 34 studies eventually eligible for the meta-analysis.^1,4^ In addition to reiterating the fact that postoperative renal dysfunction was under investigation in the present study, it ought to be borne in mind that the catecholamines can simultaneously result in lactate elevation given their actions on the glucose metabolism.^1,2^

 Even in this regard, the links between the glucose and lactate metabolism can be difficult to overlook, particularly when Shen, et al delineate a positive correlation between the peak blood glucose and the lactate levels during CPB (r = 0.312, *P* = 0.009).^2,3^ Interestingly, the abovementioned finding originated from a retrospective analysis of the dataset of 200 non-diabetic patients undergoing CABG.^3^ Considering a more heterogenous cohort of diabetics and non-diabetics was involved in the Hadipourzadeh, et al study, they should have notified their blood glucose management, especially when evaluating lactate as a closely related parameter.^1^

 Lastly, it is humbly registered that the predisposition to renal dysfunction in the index study, could have been more comprehensively addressed. Employing meta-analytical evidence by Yi, et al emerging from 2,157 cases and 49,777 controls as an example, the role of baseline risk-factors such as peripheral vascular disease and for that matter, complications like low cardiac output syndrome cannot be undermined when studying AKI as an outcome of interest.^1,5^ It is also believed that the glycated hemoglobin levels (HbA1c) could have been presented for the diabetic cohort in the Hadipourzadeh et al study, when independent researchers hint towards a heightened AKI-risk in those with HbA1c more than 7%.^1,6^

## Competing Interests

 Nothing to declare, including no use of artificial intelligence in drafting.

## Ethical Approval

 Not applicable.
